# Body temperature abnormalities in non-neurological critically ill patients: a review of the literature

**DOI:** 10.1186/2052-0492-2-14

**Published:** 2014-02-18

**Authors:** Shigeki Kushimoto, Satoshi Yamanouchi, Tomoyuki Endo, Takeaki Sato, Ryosuke Nomura, Motoo Fujita, Daisuke Kudo, Taku Omura, Noriko Miyagawa, Tetsuya Sato

**Affiliations:** Division of Emergency and Critical Care Medicine, Tohoku University Graduate School of Medicine, Seiryo-machi 2-1, Aoba-ku, Sendai, Miyagi, 980-8574 Japan; Department of Emergency and Critical Care Medicine, Tohoku University Hospital, Seiryo-machi 1-1, Aoba-ku, Sendai, 980-8574 Japan

**Keywords:** Critical care, Fever, Hypothermia, Severe sepsis

## Abstract

Body temperature abnormalities, which occur because of several infectious and non-infectious etiologies, are among the most commonly noted symptoms of critically ill patients. These abnormalities frequently trigger changes in patient management. The purpose of this article was to review the contemporary literature investigating the definition and occurrence of body temperature abnormalities in addition to their impact on illness severity and mortality in critically ill non-neurological patients, particularly in patients with severe sepsis. Reports on the influence of fever on outcomes are inconclusive, and the presence of fever *per se* may not contribute to increased mortality in critically ill patients. In patients with severe sepsis, the impacts of elevated body temperature and hypothermia on mortality and the severity of physiologic decline are different. Hypothermia is significantly associated with an increased risk of mortality. In contrast, elevated body temperature may not be associated with increased disease severity or risk of mortality. In patients with severe sepsis, the effect of fever and fever control on outcomes requires further research.

## Introduction

Body temperature is a routinely measured vital sign in all patients admitted to the intensive care unit (ICU). Body temperature abnormalities are among the most commonly noted symptoms of critically ill patients, and they occur because of several different infectious and non-infectious etiologies. Moreover, these abnormalities frequently trigger changes in patient management [1, 2].

Fever occurs in approximately half of the patients admitted to the ICU and is reported to be associated with adverse outcomes [3], although its incidence in adult patients in the ICU may decrease in recent years [4]. Fever is part of a host’s acute phase response to infectious as well as non-infectious inflammatory stimuli [5] and, as such, is one of the most prominent symptoms of infection [2]. Fever is believed to be harmful, especially in patients with life-threatening illnesses, because febrile responses are known to increase the metabolic rate, minute ventilation, and oxygen consumption; therefore, it can have adverse effects on neurological outcomes [6–8]. Conversely, the beneficial effects of fever may include reduction in bacterial growth and promotion of antibody and cytokine synthesis, thereby activating immune cells and improving survival [9–11]. Several studies have also suggested that suppression of the febrile response with antipyretic drugs could worsen patient outcomes [12, 13].

Hypothermia can be caused by a number of factors, including cold exposure, severe infection, endocrine abnormalities, and drug overdose, all of which require intensive care [14–16]. Inflammation-associated hypothermia is considered a thermoregulatory ‘failure’ [17], and it has been recognized as a significant clinical condition. Although hypothermia may be an unintended consequence of critical illness in patients with infectious and non-infectious conditions, the influence of hypothermia on the physiological severity and outcome of critically ill patients is not well understood [18–25].

The purpose of this article was to review the contemporary literature investigating the definition and occurrence of body temperature abnormalities in addition to their impact on the disease severity and mortality in critically ill non-neurological patients, especially in patients with severe sepsis.

A literature review was conducted using PubMed (Bethesda, MD, USA) from 1966 to November 30, 2013, to identify articles focusing on the epidemiology of temperature abnormalities, their impact on severity and mortality, and their management in the ICU. Search terms included fever, hyperthermia, hypothermia, temperature, and ICU. Abstracts were screened for relevance, and appropriate full-length articles were retrieved for appraisal. The reference lists of significant articles were also screened for other pertinent articles.

## Review

### Normal body temperature and fever definition

Although it varies over the course of the day, normal body temperature is approximately 37.0°C (98.6°F) and is controlled in the thermoregulatory center of the anterior hypothalamus [26]. A normal variation of 0.5°C (0.9°F) occurs within individuals depending on the time of day [27]. Based on a study conducted in 148 healthy adults, a morning temperature of >37.2°C or an afternoon temperature of >37.7°C was considered as a fever [28]. Oral temperatures from more than 700 measurements in these adults ranged from 35.6°C to 38.5°C with a mean of 36.8°C ± 0.4°C [28]. Lower levels occurred at 6 a.m., and higher levels occurred between 4 and 6 p.m. The 99th percentile for healthy adults was indicated by the maximum normal oral temperature of 37.2°C at 6 a.m. and 37.7°C at 4 p.m., thus providing the basis for fever cutoffs, which differed based on time of day.

Because of this variability and given that the magnitude and significance of an elevated temperature will depend on the specific patient population, a wide range of definitions for fever have been reported in the literature, and there is currently no consensus. Although a core body temperature of 38.0°C is used as the cutoff value for fever in several definitions [26, 29, 30], a core temperature of 38.3°C (101°F) may be more generally accepted to represent fever in ICU patients, and this temperature is recommended in the guidelines for the evaluation of a new fever in critically ill adult patients [2].

An exact temperature measurement is critical to patient management. Oral thermometers are impractical, and axillary temperature measurements are not recommended in critically ill patients, which significantly underestimate true temperature [2]. Therefore, in the ICU, temperature is measured using a number of different methods, including thermistors on intravascular, bladder, esophageal, or rectal probes, in addition to infrared tympanic membrane and temporal artery thermometers. Although the pulmonary artery catheter has been considered the ‘gold standard’ measurement technique, in most situations, relatively small differences exist between the other commonly used methods [2, 31].

### Fever and hyperthermia

The major causes of abnormally elevated temperatures in critically ill patients can be broadly classified as infectious fevers, non-infectious fevers, and hyperthermia syndromes [1, 32]. Infectious etiologies of fever include bacterial, viral, fungal, parasitic, and protozoal types. Bacterial infections are the most common etiology and are typically associated with positive cultures. The most common sites of bacterial infection in critically ill patients are the lower respiratory tract, urinary tract, primary bloodstream, and intra-abdominal region [33–36]. Non-infectious causes of fever are also common and include myocardial infarction, pancreatitis, drug hypersensitivity reactions, transfusion reactions, venous thromboembolic disease, deep body site hematomas, and neurogenic fever such as that following subarachnoid hemorrhage. Hyperthermia syndromes include heat stroke, neuroleptic malignant syndrome, malignant hyperthermia, severe thyrotoxicosis, pheochromocytoma, and adrenal crisis [1, 37, 38].

The thermostat device, which regulates the room temperature in a home, is comparable to the way the hypothalamus controls core body temperature. The thermostat setting in the hypothalamic thermoregulatory center shifts upwards during a fever due to infectious or non-infectious causes, i.e., during fever, the ‘set point’ in the hypothalamus shifts upwards from the ‘normothermia’ setting to febrile levels. Elevated levels of prostaglandin E_2_ in the hypothalamus appear to trigger the increased set point, resulting in the activation of neurons in the vasomotor center that commence vasoconstriction and of warm-sensing neurons that slow their firing rate and increase heat production in the periphery [39].

In contrast to the actions during fever, the setting of the thermoregulatory center during hyperthermia remains unchanged at normothermic levels, while body temperature increases in an uncontrolled fashion and overrides the ability to lose heat. Exogenous heat exposure and endogenous heat production are two mechanisms by which hyperthermia can result in dangerously high internal temperatures [39].

Because there are no clear medical definitions for fever or hyperthermia in critically ill patients, the definitions used within each study in this review differed. Abnormally elevated temperatures were categorized as fever, which is controlled by the hypothalamic thermoregulatory center, or hyperthermia, in which the body temperature increases without control by the thermoregulatory center. In addition, high fever was defined as fever with markedly elevated body temperatures (e.g., >39.5°C) in several studies. Therefore, in this review, we have used these terms for elevated body temperatures based on the definition(s) in each study.

### Body temperature abnormalities and mortality in ICU patients

Reports of the impact of fever on mortality in ICU patients are inconsistent; some studies indicate that fever may contribute to mortality, while a recent meta-analysis has suggested that the presence of fever *per se* may not increase mortality [40]. Peres Bota et al. reported that patients with fever had significantly increased mortality compared to patients with normothermia (35.3% vs. 10.3%, *P* < 0.01) in a prospective study of fever among 493 adult ICU patients [21]. Fever (core temperature ≥38.3°C) in 139 (28.2%) patients was primarily present at ICU admission (76.3%) and was of infectious origin (55%). The most common non-infectious cause of fever was postoperative fever (19%). Circiumaru et al. prospectively studied fever (core temperature ≥38.4°C) in 100 consecutive admissions of 93 patients over a 4-month period and found fever among 70% of admissions [41]. There were similar proportions of infectious and non-infectious etiologies, and most fevers lasted <5 days. The presence of prolonged fever (>5 days) was associated with increased mortality (62.5% vs. 29.6% for prolonged fever and fever, respectively, *P* < 0.0001).

Laupland et al. studied fever in 20,466 critically ill adult patients with and without infection from 2000 to 2006 [3]. The cumulative incidences of fever (core temperature ≥38.3°C) and high fever (core temperature ≥39.5°C) were 44% and 8%, respectively. Positive bacterial cultures were associated with 17% and 31% of the fever and high fever episodes, respectively. Although the presence of fever was not associated with increased mortality, the presence of high fever was associated with a significantly increased risk of death (12% vs. 20.3%, respectively, *P* < 0.0001). It was suggested that high fever could result in complications such as cardiac arrhythmias, tachycardia, increased oxygen demand, convulsions, and brain damage [3].

Regarding the association of fever with mortality in ICU patients, the impact of fever is inconsistent, and the presence of fever itself may not contribute to the increase in mortality as suggested in a recent meta-analysis [40]. However, more specifically, high fevers (≥39.5°C) and prolonged fevers (>5 days) may be associated with an increased risk of mortality [40, 42].

Comparatively, limited attention has been given to hypothermia, which has also been associated with an increased risk of mortality in critically ill patients [18, 20, 21, 43]. Laupland et al. evaluated 10,962 ICU patients, and 10% of the patients had mild hypothermia (35.0°C–35.9°C), 5% had moderate hypothermia (32°C–34.9°C), 1% had severe hypothermia (<32°C), 21% patients had a mild fever (38.3°C–39.4°C), and 5% had a high fever (>39.5°C) at presentation. Normothermia was present in 6,133 patients (55%). The overall mortality in these ICU patients was 18%: 14% with normothermia, 22% with mild hypothermia, 38% with moderate hypothermia, 60% with severe hypothermia, 18% with mild fever, 21% with high fever, and 30% with mixed temperature abnormalities. Although fever at presentation was not associated with a significantly increased risk of death, hypothermia was an independent predictor for death in medical ICU patients [43]. Therefore, hypothermia may be a major and potentially modifiable factor associated with an increased risk of death in critically ill patients.

### Body temperature abnormalities in patients with severe sepsis

Fever may not be always associated with an increased risk of mortality in patients with sepsis. A recent retrospective study with data from Australia, New Zealand, and the UK reported that an elevated peak body temperature in the first 24 h after ICU admission was associated with decreased in-hospital mortality in patients with infection [18]. The lowest mortality risk was among patients with a temperature between 39°C and 39.4°C. However, mortality risk was increased among patients with the same temperature range who did not have infection.

Similarly, the Fever and Antipyretic in Critically Ill Patients Evaluation (FACE) study observed a trend for decreased 28-day mortality in septic patients with temperatures ≥39.5°C, whereas the opposite was demonstrated for non-septic patients with temperatures ≥39.5°C [44]. Swenson et al. prospectively analyzed 823 adult surgical ICU patients with sepsis related to bloodstream infections between 1996 and 2005 in which fever was defined as a temperature ≥38.5°C [45]. Death occurred in 148 patients with bloodstream infections (18.0%), and 541 (65.7%) patients were febrile at diagnosis. The mortality in the patients with and without fever was 12.9% and 27.7%, respectively (*P* < 0.0001). A higher maximum temperature was protective against mortality (OR = 0.60, *P* < 0.0001). As a result, the authors suggested that fever during a bloodstream infection improves survival in surgical patients with sepsis.

In contrast, hypothermia may be associated with an increased risk of mortality in patients with severe sepsis [18, 20, 21, 43] as evidenced by previous large trials (Table 1) [16, 19, 46–48]. The incidence of hypothermia (<35.5°C) was 9% in the Methylprednisolone Severe Sepsis Study, 10% in the Veterans Administration Systemic Sepsis Cooperative Study of Glucocorticoid Therapy, and 9.6% in the Ibuprofen Sepsis Study, and all of these studies only included patients with severe sepsis. The incidence of 28- or 30-day mortality in patients with hypothermia compared to those without hypothermia in these studies was 62% vs. 26%, 57% vs. 28%, and 70% vs. 35%, respectively. The incidence of hypothermia in the NORASEPT II Study, in which only patients with septic shock were included, was 21%. The mortality in patients with hypothermia and in those without hypothermia was 59% and 34%, respectively. The findings of the recent study that we conducted are also consistent with these results (Table 1) [49]. In summary, hypothermia may complicate severe sepsis in approximately 10% to 20% of patients and could be associated with a risk of mortality that is twice that of non-hypothermic patients.

**Table 1:** **Incidence of hypothermia (body temperature** <**35.5°C) and associated outcomes in patients with severe sepsis**

Hypothermia (<35.5°C), %	Mortality (%)	
	<35.5°C	≥35.5°C
Methylprednisolone Severe Sepsis Study [16] (*n* = 382), 9%	62	26
Veterans Administration Systemic Sepsis Cooperative Study of Glucocorticoid Therapy [47] (*n* = 223), 10%	57	28
Ibuprofen Sepsis Study [19] (*n* = 453), 9.6%	70	35
NORASEPT II Study (tissue factor pathway inhibitor) [46], (*n* = 930, septic shock), 21%	59	34
JAAM Sepsis Registry [49] (*n* = 624), 15.9%	40.4	17.7

*JAAM* Japanese Association for Acute Medicine.

Although there are several reports of body temperature abnormalities in patients with sepsis, there is a relative paucity of information on the influences of hyperthermia or hypothermia on disease severity and outcomes in patients with severe sepsis. We investigated the association between body temperature, disease severity, and patient outcomes in patients with a definitive diagnosis of severe sepsis in a prospective, multicenter, observational study [49]. Six hundred and twenty-four patients with severe sepsis were grouped according to their core body temperature into six categories based on the temperature data of the Acute Physiology and Chronic Health Evaluation II (APACHE II): ≤35.5°C, 35.6°C–36.5°C, 36.6°C–37.5°C, 37.6°C–38.5°C, 38.6°C–39.5°C, and ≥39.6°C. Patients with temperature of ≤36.5°C had significantly worse Sequential Organ Failure Assessment (SOFA) scores when compared with patients with temperature >37.5°C on the day of enrollment. Although mortality did not relate to body temperature ranges of ≥37.6°C as compared to the reference range of 36.6°C–37.5°C, the relative risk for 28-day mortality was significantly greater in patients with 35.6°C–36.5°C and ≤35.5°C (odds ratio 2.032 and 3.096, respectively) (Table 2). When the patients were divided into groups based on the presence (≤36.5°C, *n* = 160) or absence (>36.5°C, *n* = 464) of hypothermia, those with hypothermia had worse physiological severity and significantly higher 28-day and hospital mortality rates than those without hypothermia (Table 3). The presence of hypothermia was an independent predictor of 28-day mortality, and the differences between the patients with and without hypothermia were observed irrespective of the presence of septic shock.

**Table 2:** **Body temperature of ICU admission and 28-day mortality (adapted from** [49]**)**

Range of body temperature (°C)	28-day mortality (%)	Unadjusted odds ratio	95% confidence interval	***P*** value
≤35.5	40.4	3.096	1.611–5.947	0.001
35.6–36.5	34.4	2.032	1.009–4.088	0.047
36.6–37.5	20.5	1	Reference	
37.6–38.5	18.1	0.853	0.461–1.577	0.621
38.6–39.5	15.8	0.726	0.377–1.395	0.404
≥39.6	17.2	0.803	0.363–1.778	0.693

**Table 3:** **Characteristics and outcomes in patients with severe sepsis, with and without hypothermia**

	Hypothermia (***n*** = 160)	Non-hypothermia (***n*** = 464)	***P*** value
Septic shock	59.4% (*n* = 95)	40.3% (*n* = 187)	<0.001
SOFA score	10 (7–13)	8 (5–11)	<0.001
APACHE II score	26 (21–32)	21 (16.25–27)	<0.001
Outcome			
28-day mortality	38.1% (*n* = 61)	17.9% (*n* = 83)	<0.001
Hospital mortality	49.4% (*n* = 79)	22.6% (*n* = 105)	<0.001

Hypothermia is defined as body temperature ≤36.5°C. *SOFA* Sequential Organ Failure Assessment, *APACHE* Acute Physiology and Chronic Health Evaluation (adapted from [49]).

### Treatment of fever in critically ill patients and patients with severe sepsis

Several studies have suggested that the suppression of the febrile response with antipyretic drugs could worsen patient outcomes; however, this conclusion is based on clinical trials that were of insufficient sample sizes to detect differences in mortality [12, 13, 50–52]. A meta-analysis by Hammond and Boyle demonstrated that in critically ill patients, including those with neurological injury, newer methods of physical cooling and continuous infusions of antipyretic pharmacotherapy more effectively lowered temperature than conventional physical cooling and bolus dosing of pharmacologic antipyretic therapy, respectively [53]. Another meta-analysis demonstrated that antipyretic therapy does not significantly impact mortality in septic patients (pooled OR 1.08, 95% CI 0.6–1.96) [54]. Although antipyretic therapy in critically ill adult patients may be safe and feasible [55], the impact of temperature control on the mortality of febrile critically ill patients is still unknown.

Conflicting results have been reported by recent studies investigating mortality in relation to fever control in patients with sepsis by using antipyretic treatment or external cooling [44, 56]. In the FACE study, the independent association of fever and the use of antipyretic treatments on mortality in critically ill non-neurological patients with and without sepsis (*n* = 1,425) was investigated. They reported that treatment with non-steroidal anti-inflammatory drugs (NSAIDs) or acetaminophen independently increased 28-day mortality in septic patients (NSAIDs: adjusted OR 2.61, *P* = 0.028; acetaminophen: adjusted OR 2.05, *P* = 0.01), but not in non-septic patients [44]. Fever control by external cooling for vasopressor requirements in septic shock has been evaluated in a multicenter, randomized, controlled trial [56]. Febrile patients with septic shock requiring vasopressors, mechanical ventilation, and sedation were allocated to external cooling (*n* = 101) to achieve normothermia (36.5°C–37°C) for 48 h or no external cooling (*n* = 99). The primary endpoint was the number of patients with a 50% decrease in the baseline vasopressor dose after 48 h. A decrease in the vasopressor dose was significantly more common with external cooling after 12 h of treatment (54% vs. 20%; absolute difference, 34%; 95% CI −46 to −21, *P* < 0.001). Shock reversal during the ICU stay was significantly more common with cooling, and the cooling group had significantly lower 14-day mortality (19% vs. 34%; absolute difference, −16%; 95% CI −28 to −4, *P* = 0.013). Therefore, fever control using external cooling may decrease vasopressor requirements and early mortality during septic shock. However, further research is required to elucidate the role of fever and its control in patients with severe sepsis [57].

## Conclusions

Body temperature abnormalities are among the most commonly noted symptoms in critically ill patients. Reports of the influence of fever on the outcomes in these patients are inconsistent, and the presence of fever *per se* may not contribute to increased mortality in critically ill patients. In patients with severe sepsis, the impacts of elevated body temperature and hypothermia on mortality and the severity of physiologic decline are different. Elevated body temperature alone may not influence disease severity or mortality; however, hypothermia has been associated with a significant increase in mortality risk. Further research regarding the role of body temperature abnormalities in the risk of mortality is warranted.

## Abbreviations

ICU: Intensive care unit
FACE: Fever and Antipyretic in Critically Ill Patients Evaluation
APACHE: Acute Physiology and Chronic Health Evaluation
SOFA: Sequential Organ Failure Assessment
NSAIDs: Non-steroidal anti-inflammatory drugs.
